# Pathogenesis of chronic rhinosinusitis with nasal polyp and a prominent T2 endotype

**DOI:** 10.1016/j.heliyon.2023.e19249

**Published:** 2023-08-23

**Authors:** Said Ahmad Shah, Masayoshi Kobayashi

**Affiliations:** Department of Otorhinolaryngology-Head and Neck Surgery, Mie University Graduate School of Medicine, Mie, Japan

**Keywords:** Chronic rhinosinusitis, Eosinophilia, Nasal polyp, Monoclonal antibody, T cell response

## Abstract

Chronic rhinosinusitis is a heterogenous and multifactorial disease, characterized by persistent inflammation of the nose and paranasal sinuses, which causes nasal obstruction, nasal discharge, facial pain, and smell disturbance. Chronic rhinosinusitis is divided into two phenotypes: chronic rhinosinusitis with nasal polyp and chronic rhinosinusitis without nasal polyp. Nasal polyps can be associated with many inflammatory cells including eosinophil cells, neutrophil cells, plasma cells, and lymphocytes. T2 endotype is characterized by the type-2 immune response and nasal polyps are associated with eosinophilic dominant infiltration. In contrast, in the T1 and T3 endotypes, chronic rhinosinusitis can be associated with neutrophilic dominant infiltration. In addition, there are mixed types of inflammation with different proportions of eosinophils-neutrophils in chronic rhinosinusitis. In the T2 endotype, there is an increase in the production of Th2 cytokines, including interleukin-4, interleukin-5, and interleukin-13, high levels of immunoglobulin-E in polyp tissue, and eosinophilia. Stimulation of Th2 cells, type-2 innate lymphoid cells, epithelial cell damage, Staphylococcus aureus enterotoxins, and autoimmune antibodies have important roles in the enhancement of Th2 cytokines and pathogenesis of chronic rhinosinusitis with nasal polyp. Monoclonal antibodies target type-2 inflammation, decrease nasal polyp size, and improve the clinical symptoms of CRSwNP patients. The present review will focus on factors involved in the pathogenesis of chronic rhinosinusitis and its treatment.

## 1 Introduction

Chronic rhinosinusitis (CRS) is characterized by persistent inflammation of the nasal and paranasal sinus mucosa with the presence of at least two of the cardinal symptoms: either nasal obstruction and/or nasal discharge with smell problems or facial pain or pressure for at least 12 weeks [1]. CRS is associated with a significant impairment of quality of life (QOL) [2]. A recent study revealed that among the principal rhinologic symptoms, headache, nasal congestion, and hyposmia had the most impact on the QOL of the patients though hyposmia was rarely spontaneous [3].

Two types of CRS are commonly described: CRS with nasal polyps (CRSwNP) and CRS without nasal polyps (CRSsNP). These two types have different clinical and morphological characteristics [4]. CRSwNP is characterized by type-2 inflammation, is often severe and recurrent, and presents with comorbidities such as N-ERD (NSAID-exacerbated respiratory disease) and asthma [5]. The recurrence rate is more than half in patients of CRSwNP with type-2 inflammation who have significantly higher eosinophilia, particularly in those who have 10% or higher peripheral blood eosinophils [6,7].

In Western countries, CRSwNP is characterized by predominant eosinophilic inflammation, whereas CRSsNP exhibits predominant Th1 characteristics. In contrast, in Japan and East Asia, neutrophilic inflammation has been predominant, while recently CRSwNP with eosinophilic infiltration has increased [6]. CRSwNP and asthma share similar pathophysiology and are associated with eosinophilic infiltration and Th2 cytokines such as interleukin-4, interleukin-5, and interleukin-13 (IL-4, IL-5, and IL-13) as well as high levels of local immunoglobulin-E (IgE) [8].

CRSwNP with asthma is associated with severe symptoms, and asthma with nasal polyposis is prone to more exacerbation and is difficult to control with medical and surgical treatments [9]. Th1 and Th2 cell balance is dysregulated in CRS [10].

Following asthma, endotypes have been established in CRS. Endotyping is useful for the prediction of the natural course of CRS, and to decide pharmacotherapy and surgery as well as selecting patients for treatment with biologics [11].

Traditionally T2 endotype was considered to be associated with a type-2 inflammatory profile and eosinophilic infiltration in CRSwNP patients, whereas CRSsNP patients could show T1 and T3 endotypes and neutrophilic infiltration. However, recent studies revealed a mixed type of eosinophilic-neutrophilic inflammation with severe cases of type-2 immune response in CRSwNP patients. On the other hand, CRSsNP patients have shown significantly increased and activated eosinophils and a type-2 immune response [6,12].

Several factors are involved in the pathogenesis of CRS that promote nasal polyp formation and the type-2 immune response resulting in the T2 endotype that is the focus of this study.

## 2 Th1, Th2, and Th17 cells

CRSwNP and CRSsNP have different morphological characteristics [4]. Histologically, CRSwNP is accompanied by eosinophil and neutrophil infiltration [13]. Nasal polyps with neutrophils infiltration are accompanied by Th1, Th17, and expression of IL-17A, tumor growth factor- β1 (TGF-β1), interferon-γ (IFN-γ), and IL-22 [10,14].

TGF-β1 plays a considerable role in the formation and growth of nasal polyps and remodeling, and TGF-β1 and IFN-γ both induce epithelial-mesenchymal transition (EMT) [15,16]. The expression of IL-17A mRNA is significantly higher in non-eosinophilic chronic rhinosinusitis (non-ECRS) polyps than in ECRS polyps and control group in Japanese patients and was increased in a group of Chinese CRSwNP patients who had low levels of IL-5 [10,17].

IL-17A mediates neutrophils recruitment to the site of inflammation and there is a positive correlation between IL-17A and neutrophil counts in the airway [18]. Nonetheless, IL-17A has been reported to have an important role in goblet cell hyperplasia and MUC5AC production in ECRS [19].

A high level of IL-8 has been observed in neutrophil-dominant polyps in the Japanese population. In addition, mast cells, tissue plasminogen activator, and factor XIII‐A play an important role in promoting polyp formation and disease prognosis, particularly after ESS [6]. Based on these observations, non-ECRSwNP associated with Th1/Th17 activation is more prevalent in Asian countries [20].

Nasal polyps with eosinophils infiltration are accompanied by type-2 inflammation expressing IL-4, IL-5, and IL-13 and high levels of circulatory but particularly local IgE [21]. These cytokines are key and central drivers of type-2 inflammation, activating and recruiting mast cells, eosinophils [22], basophils, goblet cells, M2 macrophages, and B cells, and they are responsible for many inflammatory tissue responses [9]. Generally, CRSsNP is characterized mainly by neutrophilic inflammation with elevated levels of Th1 cytokines, whereas CRSwNP is often associated with eosinophilic inflammation and increased levels of Th2 cytokines [23]. However, Japanese patients have shown increased levels of Th1 cytokine with prevalent neutrophilic infiltration in CRSwNP [10]. Nonetheless, recent studies revealed a considerable changing pattern of CRSwNP throughout Asia with increased eosinophilic infiltration and comorbid asthma in patients with CRSwNP [24].

The degree of mucosal eosinophil infiltration provides prognostic information for the diagnosis and disease severity. According to the JESREC (Japanese Epidemiological Survey of Refractory Eosinophilic Chronic Rhinosinusitis), there was a significant correlation between mucosal eosinophilia of 70 or higher eosinophils/HPF with recurrence of CRS after ESS and both higher mucosal eosinophils infiltration of the submucosa of the ethmoid cavity or nasal polyp are of diagnostic value [6].

## 3 Regulatory T cells (tregs)

Treg cells are a subpopulation of T cells that act to regulate the immune response. Treg cells have an important function in the T cell subtype regulation and expression in patients with CRSwNP both in Caucasians and Asians [10]. Treg cells secrete IL-10 and TGF-β [25]. Forkhead box P3 (FOXP3) expression is essential for Treg cell development, stability, and function [26]. On the other hand, GATA3 (GATA-binding protein 3) is essential for the maintenance of Foxp3 expression [27] and promotes the accumulation of Treg cells [28].

The expression of GATA3 mRNA and IL-10 is reduced in ECRS, whereas the expression of FOXP3, IL-10, and TGFβ is significantly increased in non-ECRS [10,28]. Moreover, the GATA3 transcription factor has an important role in the development and proliferation of Th2 cells and controls the expression of IL-4 and IL-5 [10,25]. Treg cells control the balance between Th1 and Th2 cells and reduce eosinophils recruitment in CRSwNP, however, the function of Treg cells seems to be defective in CRSwNP [10,29].

## 4 Group 2 innate lymphoid cells (ILC2s)

ILC2s are significantly enhanced in the nasal tissue of patients with ECRS compared to non-ECRS, CRSsNP, and controls [30]. A recent study reported a 100-fold elevation of ILC2s in nasal polyps compared with the sinus mucosa of controls [31]. Activated ILC2s express transcription factor GATA3 and produce Th2 cytokines [32]. ILC2s are activated by antigen-independent mechanisms and various factors including IL-25, IL-33, and thymic stromal lymphopoietin (TSLP) play important roles in their activation [31].

Activated ILC2s by IL-25, IL-33, and TSLP lead to eosinophilia, mucus hypersecretion, and remodeling of mucosal tissues [33]. Moreover, ILC2s are induced and activated by lipid mediators including prostaglandin D2 (PGD2) and cysteinyl leukotrienes (CysLT) (leukotriene C4 (LTC4), leukotriene D4 (LTD4), and leukotriene E4 (LTE4) promoting the production of IL-4, IL-5, and IL-13 [32,34]. IL-5 promotes eosinophilia, and IL-4 and IL-13 enhance the production of eosinophil chemoattractants (CCL11/eotaxin-1, CCL24/eotaxin-2, and CCL26/eotaxin-3) and activate B cells, macrophages, fibroblasts, epithelial cells, and goblet cells. The activation of these cells results in IgE-mediated reactions, eosinophils recruitment, mucus production, remodeling, and fibrosis [31,32,35].

Furthermore, ILC2s are activated by RANK-L mainly expressed in Th2 cells and CXCL16+ DCs, in nasal polyps [36]. ILC2s have an important role in type-2 immune response in CRSwNP [37]. Evaluation of various functions of ILC2s will contribute to novel findings in the pathogenesis of CRS and nasal polyps.

## 5 Nasal epithelium

The production of epithelial cytokines IL-25, IL-33, and TSLP is induced in response to epithelial damage by pathogens, proteases, allergens, and irritants [38,39]. These cytokines promote the development of Th2 cytokines (IL-4, IL-5, and IL-13) and eosinophilic inflammation in CRSwNP [40]. IL-25, IL-33, and TSLP activate both ILC2s and Th2 cells in CRSwNP increasing the production of Th2 cytokines [37,41].

Among the cytokines produced by damaged epithelium, TSLP is the most highly expressed in CRS tissue activating ILC2s and mast cells resulting in a considerable quantity of Th2 cytokines production [38]. Recent studies also report that in damaged tissue, IFN-γ upregulates IL-33 which in turn contributes to switching Th1/Th17 to a Th2 inflammation in CRSwNP [42]. IL-25, IL-33, and TSLP contribute to promoting Th2 cytokines, in addition to their direct effects on CRSwNP.

## 6 Infection

The role of bacteria and fungi is important in CRS. In allergic fungal rhinosinusitis (AFS) there is a Type-2 immune response to a fungal antigen often related to Aspergillus [43]. Among bacteria, *Staphylococcus aureus,* a gram-positive organism is the most frequently isolated species in CRS [44]. *Staphylococcus aureus* colonization is reported to be higher in the nasal cavity of patients of CRSwNP compared to CRSsNP and healthy subjects [45].

*Staphylococcus aureus s*ecret enterotoxins (Toxic shock syndrome toxin-1 (TSST-1), Staphylococcus aureus enterotoxin A and B (SEA and SEB)) and causing stimulation of T cells via their superantigen activity [46]. *Staphylococcus aureus* superantigens contribute to the polarization of type-2 immune response in nasal polyp tissue characterized by Th2 cytokines (IL-4, IL-5, IL-13) production and promote eosinophils infiltration [47]. In addition, Staphylococcus aureus enterotoxins cause IgE production through B cells and T cells activation and the release of inflammatory mediators [48].

Moreover, protease produced by *Staphylococcus aureus as well as other bacteria, fungi, viruses, mites, and pollen breakdown the barrier function of epithelial cells* [49]*. It induces the production of Th2-promoting cytokines including* IL-25, IL-33, and TSLP, and plays an important role in the pathogenesis of nasal polyp and CRS [9]. Fortunately, cystatin A and SPINK5 (Serine Peptidase Inhibitor Kazal Type 5) significantly inhibit the secretion of IL-25, IL-33, and TSLP [49].

Toll-like receptors (TLRs) contribute to airway disease and their expression level and function are altered in response to viral and bacterial particles [50]. TLR2 and TLR4 are shown to be increased in CRSwNP epithelial cells compared to normal nasal epithelial cells [47]. SEB decreases epithelial barrier integrity and promotes IL-6 and IL-8 production and mediates the disruption of tight junctions (TJs) on activation of TLR2 signaling [47]. Thus, Staphylococcus *aureus* has an important role in the induction of Th2 immune response and pathogenesis of nasal polyp and CRS.

## 7 Autoimmunity and complement fixation

Recent studies suggest there is B cell dysregulation, increased local class switching, increased antibodies, and the presence of autoantibodies in CRSwNP [51]. Elevation of immunoglobulins including total and specific IgA, IgD, IgM, IgG, and IgE levels manifested in CRSwNP could explain the autoreactive nature [[51], [52], [53]].

A recent study reported increased levels of IgG and IgA in both eosinophilic and non-eosinophilic nasal polyps, whereas elevated levels of IgE and IgD were observed in eosinophilic nasal polyps [54]. Moreover, nasal polyps secret dsDNA-specific IgG and IgA, and elevated levels of anti-dsDNA IgG and IgA antibodies are found in nasal polyps, particularly in patients requiring repeated surgeries for recurrence of nasal polyps [4,55,56]. IgE and IgD activate mast cells in local eosinophilic inflammation and IgG participates in the activation of classical complement pathways in nasal polyp tissue [54]. The complement system is one of the fundamental parts of innate and adaptive immune systems and has an important role in increasing the ability of antibodies against infection and eliminating injured, apoptotic, and aberrant cells [57].

A recent study has reported that complement activation is elevated in CRSwNP tissue and suggested an important role of the classical antibody-mediated complement pathway in complement activation in nasal polyps [51]. Elevated levels of intracellular stores of C3 and C3a have been demonstrated in CRSwNP HSNECs (Human sinonasal epithelial cells), whereas C3aR deficiency and C3aR antagonism have been shown to reduce sinonasal inflammation [58]. Split products of C3 lead to Th2 reaction in asthma and allergy, and C3a recruits eosinophils, whereas both C3a and C3b activate eosinophils [59].

**Fig. 1:**
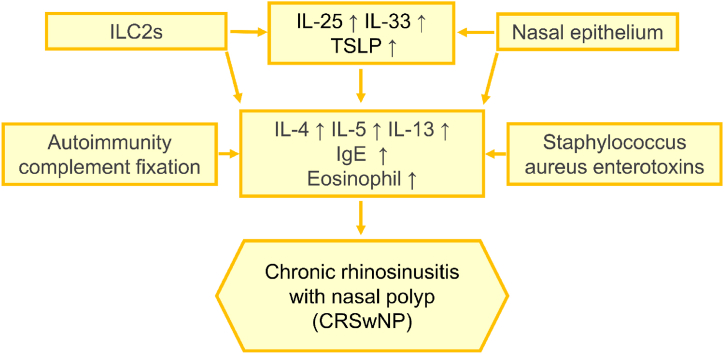
Factors involved in the pathogenesis of CRSwNP. Damage in the nasal epithelium, Th cells, ILC2s, Staphylococcus aureus enterotoxins, and autoimmunity/complement fixation causing an increase in overexpression of IL-4, IL-5, IL-13, and eosinophilia and consequently Th2 immune response, and CRSwNP.

**Fig. 2:**
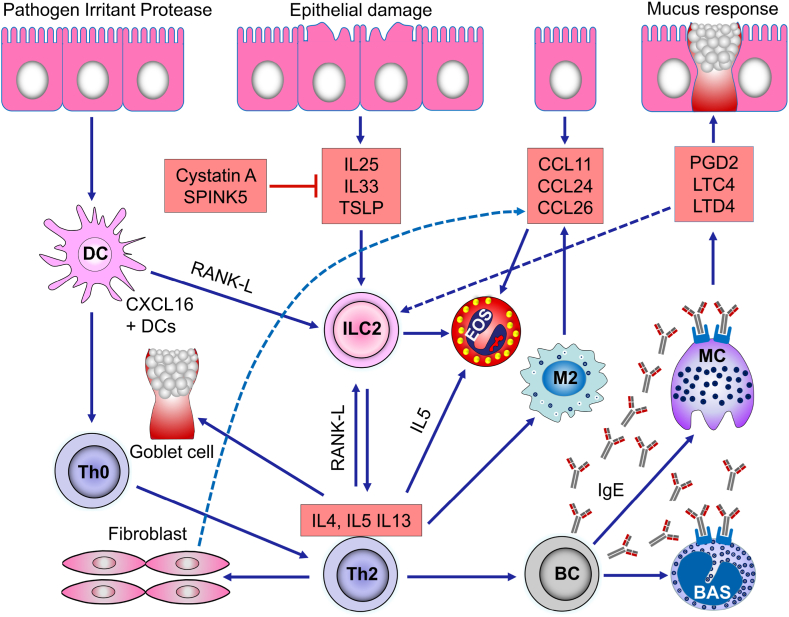
Type-2 inflammation pathway in chronic rhinosinusitis. Antigen-presenting cells including dendritic cells activate Th2 cells to produce Th2 cytokines including IL-4, IL-13, and IL-5. Staphylococcus aureus enterotoxins, damaged epithelium, and ILC2s lead to the IL-25, IL-33 and TSLP production that promote Th2 cytokines production and eosinophils infiltration. IL-5 is produced by Th2 cells and CCL11, CCL24, and CCL26 are produced by M2 (macrophage), fibroblasts, and epithelial cells promoting eosinophils. IL-4 and IL-13 activate fibroblast, goblet cell, and mucus responses. IL-4 and IL-13 promote B cells leading to the IgE switching and synthesis. PGD2, LTC4, and LTD4 are released by mast cells through IgE and mast cell reactions. PGD2, LTC4, and LTD4 affect ILC2s and nasal epithelium. ILC2s are also activated by IL-25, IL-33, and TSLP from epithelial cells, and RANK-L is expressed in CXCL16+ DCs and Th2 cells. IgE also activates basophils.

Furthermore, recent studies show that the B cell population contains high levels of intracellular C3, and activated B cells produce anti-basement membrane autoantibodies that have an important role in complement activation and epithelial damage [60]. Understanding the mechanisms involved in autoantibodies and complement fixation would provide more options for potential treatment. Sources of cytokines are summarized in (Table 1), factors involved in the pathogenesis of CRSwNP are shown in (Fig. 1), and the inflammatory pathways related to CRSwNP are shown in (Fig. 2).

**Table 1:** Shows the sources of the secretions that probably lead to the T1, T2, and T3 endotypes.

Source	Secretion	Consequence
Th1 cells	IFN-γ	T1 endotype
Th2 cells	IL-4, IL-5, IL-13	T2 endotype
Th17 cells	IL-17	T3 endotype
Treg cells	IL-10, TGFβ	Th1/Th2 balance
ILC2s	IL-25, IL33, TSLP	T2 endotype
Nasal epithelium	IL-25, IL33, TSLP	T2 endotype
Staphylococcus aureus	SEA, SEB, TSST-1, protease	T2 endotype
B cells	IgA, IgD, IgM, IgG, IgE	T2 endotype

## 8 Treatment of CRSwNP

The treatment of CRSwNP aims to control clinical symptoms and minimize the use of medications and surgical interventions [7]. Surgeries are traditionally performed when medical treatment is not effective. Several categories of medical treatments are practiced according to the clinical manifestation and diagnosis of the CRS (Table 2). Antibiotics are advised for the treatment of infection in CRS.

**Table 2:** Shows medical and surgical treatments for chronic rhinosinusitis and nasal polyp.

Treatment	Target	Disease
Omalizumab (Anti-IgE)	IgE	CRSwNP
Dupilumab (Anti-IL-4R)	IL-4, IL-13	CRSwNP
Mepolizumab (Anti-IL-5)	IL-5	CRSwNP
Reslizumab (Anti-IL-5)	IL-5	CRSwNP
Benralizumab (Anti-IL-5R)	IL-5	CRSwNP
Corticosteroids	Inflammation, T2, T1, T3	CRSwNP/CRSsNP
Antibiotics (macrolide)	Inflammation	non-ECRS
Surgery (ESS)	Nasal polyp, nasal obstruction	CRSwNP/CRSsNP

### 8.1 Monoclonal biologics

Monoclonal antibodies have been successfully used in the treatment of allergic rhinitis and asthma. Monoclonal antibodies are targeting type-2 immune reactions and are commonly used in CRS for the treatment of nasal polyps and clinical improvement [61].

Many countries have different guidelines for the indication of biologics. Some guidelines indicate all endotypes, and others suggest that confirmation of type-2 is mandatory. Nonetheless, biologics target specific molecules involved in type 2 inflammation [62].

Generally, the criteria for indication of monoclonal antibodies are evidence of type-2 inflammation, the requirement of systemic corticosteroids in the past two years, significant loss of smell and impairment of QOL, and diagnosis of comorbid asthma. Biologics could be indicated in patients who meet three of these criteria and have bilateral nasal polyps with a history of previous sinus surgery [7].

#### 8.1.1 Omalizumab (Anti-IgE antibody)

IgE is the main factor in Th2 inflammation. The numbers of B cells and plasma cells are high and the concentration of IgA, IgG, and IgE is elevated in nasal polyp tissue [63].

IgE class switching depends on IL-4, IL-13, and the expression of CD40 ligand (CD40L). IL-13 is produced by Th2 cells and IL-4 is produced by Th2 cells and mast cells, whereas the expression of CD40 is restricted to mast cells [63,64]. Omalizumab is recommended for patients with elevated IgE levels [65]. Omalizumab binds to the Cε3 domain of the four chains (Cε1-Cε4) of IgE that overlaps the binding sites for both FcεRI high affinity and FcεRII low-affinity receptors and prevents the binding of free IgE. FcεRI is expressed on mast cells and basophils and FcεRII (CD23) is expressed on APCs and B cells [63]. Omalizumab targets Th2 inflammation in nasal polyposis, decreases the size and score of the nasal polyps, and ameliorates the symptoms of CRS [48].

#### 8.1.2 Dupilumab (anti-IL−4R)

IL-4 and IL-13 are the main drivers of type-2 inflammation, involved in IgE synthesis and airway remodeling. Dupilumab is a monoclonal antibody and blocks the shared receptor (IL-4R) for IL-4 and IL-13 which has an important role in the pathogenesis of CRSwNP [66].

Dupilumab reduces the size of the nasal polyp, sinus opacification, and symptom severity and provides a rapid clinical improvement in bilateral nasal polyps and comorbid asthma in patients with CRSwNP [[67], [68], [69]]. Dupilumab is well tolerated, and interestingly, no notable pharmacokinetic difference has been demonstrated between the Japanese and non-Japanese populations other than a slightly higher concentration of dupilumab in the serum of Japanese likely as a result of the tendency toward a lower body weight [69].

#### 8.1.3 Anti-IL-5 and IL-5R (mepolizumab, Reslizumab, benralizumab)

In CRSwNP, there are tissue eosinophilia and elevated IL-5 levels. IL-5 has a crucial role in promoting eosinophils recruitment, activation, and survival. Mepolizumab is a monoclonal antibody that targets and inhibits IL-5 and decreases nasal polyp size, nasal obstruction, and symptoms of CRS [70]. Previously, a randomized double-blind placebo-controlled trial has shown that in patients with recurrent nasal polyposis receiving topical corticosteroid and needing surgery, treatment of mepolizumab reduced the need for surgery and improved symptoms compared to the placebo group [71].

Reslizumab is also suggested in patients with elevated levels of eosinophils [65]. Benralizumab is a cytotoxic monoclonal antibody that binds to the IL-5 receptor (IL-5Rα) expressed on eosinophils and basophils and blocks IL-5 signaling. Benralizumab reduces nasal polyp size and nasal blockage and improves the sense of smell in CRSwNP [72]. There are other biologics such as anti-IL-33 and vitamin D supplementation that could be candidates for the treatment and clinical improvement of CRSwNP/CRSsNP [43,73,74].

Recent studies have revealed that the level of Th2 cytokines (IL-5 and IL-13) were significantly higher in the analysis of uncinate process mucosa tissues from CRSsNP in Korean patients [75]. On the other hand, no significant difference was found in IL-4 level in controls, ECRS, and non-ECRS polyps in Japanese patients [10]. Although more research is needed, it would be interesting to use monoclonal antibodies in the T1 endotype targeting Th2 cytokines in T2 endotypes.

### 8.2 Antibiotics

Although traditionally treatment of CRSwNP included nasal saline irrigation, corticosteroids, antibiotics, and surgery, currently no recommendation is suggested to use non-macrolide antibiotics for the treatment of CRSwNP and thus a placebo-controlled study would be a research priority to evaluate the effect of antibiotics in the treatment of CRSwNP [76,77]. Macrolide has immunomodulatory and antibiotic properties [78] and it causes a reduction in IL-8 levels in human nasal epithelial cells [79].

In Japanese populations, a high level of IL-8 has been shown in neutrophil-dominant nasal polyps that also demonstrate eosinophils [6,22]. The combination of ESS with long-term-low-dose macrolide treatment controls the symptoms of the patients who exhibit non-ECRS, whereas ECRS seems to be unresponsive to macrolide therapy [6,22]. Therefore, while prescribing medicine, the selection of patients and endotype is important.

### 8.3 Steroids

Corticosteroids have been considered effective medicines for the treatment of CRSwNP.

Corticosteroids have strong anti-inflammatory effects and suppress type-2 inflammation, decrease eosinophil numbers, and reduce eosinophil cationic protein (ECP) and IL-5 levels in nasal polyp [80]. Corticosteroids alter T2 response greater than T1 and T3 defining their better roles in the treatment of CRSwNP compared to CRSsNP [66]. Initially, treatment is started with intranasal corticosteroids. Oral corticosteroids are used in uncontrolled cases of CRSwNP to reduce polyp size and improve symptoms although may have systemic side effects [9,20,61].

### 8.4 Surgery

After the failure of medical treatment, surgery is an option to clear the sinonasal passage of

patients suffering from CRSwNP. However, often patients have rapid polyp regeneration and therefore multiple revision surgeries are required [6,61]. Endoscopic sinus surgery (ESS) is performed to increase nasal airflow and sinus drainage and to provide a facility for topical medical treatment delivery [81]. CRSwNP with comorbid asthma is severe and is often characterized by a recurrent nasal polyp and requires a higher dose of corticosteroid than the patients with asthma alone [9].

ESS has shown benefits in patients of CRSwNP with comorbid asthma in reducing nasal polyp size and minimizing the need for medication in asthmatic patients [22]. Nonetheless, the ideal extent of ESS remains controversial. A recent randomized controlled trial compared the efficacy and safety of limited ESS with partial ethmoidectomy and extended ESS with total ethmoidectomy by hypothesizing that extended sinus surgery is superior to limited sinus surgery in patients with severe CRSwNP [82].

## 9 Summary and conclusion

Factors including the expression of Th cells and ILC2 cells, epithelial cell damage, Staphylococcus aureus enterotoxins, and autoimmunity have important roles in the pathogenesis of CRSwNP and establishing a T2 endotype. Elimination of these factors would help in preventing and treating CRSwNP with a prominent T2 endotype. Treatment of CRSwNP has been challenging in the past. However, recent studies and clinical trials suggest that monoclonal antibodies are effective in decreasing symptoms of CRSwNP patients. Nonetheless, the understanding of the pathogenesis of CRS is progressively developing. There are several inflammatory processes in the sinonasal cavity, type-2 with heavy eosinophils, non-type-2, and overlap of type-2 and non-type-2. Currently, biologics are used to treat type-2 CRSwNP patients. More studies are needed to investigate novel biologics that would affect different inflammatory responses of the sinonasal cavity and to follow the long-term effects of biologics and the challenges faced in endotypes.

## Author contribution statement

All authors listed have significantly contributed to the development and the writing of this article.

## Data availability statement

Data included in article/supp. material/referenced in article.

### Additional information

No additional information is available for this paper.

## Declaration of competing interest

The authors declare that they have no known competing financial interests or personal relationships that could have appeared to influence the work reported in this paper.
